# Parasitism by pinnotherid crabs in the Japanese scallop *Mizuhopecten yessoensis*: first host record and quantitative assessment of host impacts

**DOI:** 10.1016/j.ijppaw.2026.101198

**Published:** 2026-01-24

**Authors:** Tomoyasu Yamazaki, Kenji Odani, Ryo Nakayama, Tetsuya Watanabe, Souichirou Kawai

**Affiliations:** a Toyo Institute of Food Technology, 23-2, 4-chome, Minami-hanayashiki, Kawanishi, Hyogo, 666-0026, Japan; b Fisheries Research Institute, Aomori Prefectural Industrial Technology Research Center, 10 Tsukidomari, Moura, Hiranai, Aomori, 039–3381, Japan; c Nishinomiya Shell Museum, 4-13-4, Nishinomiyahama, Nishinomiya, Hyogo, 662-0934, Japan

**Keywords:** Pinnotheridae, Pea crabs, Host–parasite interaction, Japanese scallop, Aquaculture, Stage-dependent effects

## Abstract

Pea crabs of the family Pinnotheridae are widely known as symbionts or parasites of bivalve mollusks; however, their occurrence and ecological impacts remain poorly documented for many economically important host species. Here, we report the first confirmed host record of pinnotherid crabs inhabiting the mantle cavity of the Japanese scallop *Mizuhopecten yessoensis* from Mutsu Bay, northern Japan, and quantitatively assess their effects on host growth and somatic condition.

A total of 881 scallops, including juvenile and subadult individuals, were examined between March 2024 and November 2025. Pinnotherid crabs were detected across multiple localities, with parasitism prevalence of 32.8 % in juvenile scallops and 27.3 % in subadults, showing no significant difference between developmental stages. Mature females, including ovigerous individuals, were observed within host scallops, indicating successful reproduction of the crabs inside this newly documented host.

Despite similar prevalence across stages, the impacts of parasitism were strongly stage dependent. Parasitized juvenile scallops exhibited significantly reduced shell length and soft tissue index compared with non-parasitized individuals, whereas no significant effects were detected in subadults. Results from size-adjusted statistical models indicated that parasite load significantly reduced somatic condition independent of host shell length. Quantile regression further revealed a strong host–parasite size constraint, with maximum crab size increasing with host size.

These results indicate that pinnotherid crabs function as true parasites in *M. yessoensis*, particularly during early life stages, imposing measurable physiological costs. Our findings highlight parasitism as a previously overlooked biotic factor that may influence early-life performance and resilience in a major aquaculture species under environmental stress.

## Highlights

• First host record of pinnotherid crabs in the Japanese scallop *Mizuhopecten yessoensis.*
• Parasitism prevalence was similar across developmental stages.
• Parasitism markedly reduced growth and condition in juvenile scallops.
• Size-adjusted analyses revealed direct physiological costs of parasitism.

## Graphical abstract

**Figure:**
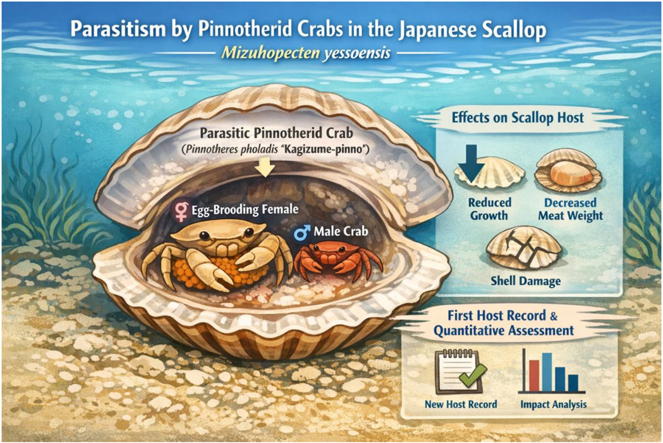

## 1 Introduction

Pea crabs of the family Pinnotheridae represent a diverse group of brachyuran crabs characterized by symbiotic associations with a wide range of marine invertebrates, particularly bivalve mollusks. Members of this family exhibit a continuum of symbiotic strategies, ranging from facultative commensalism to obligate parasitism, often inhabiting the mantle cavity or gill chambers of their hosts (De Haan.,; Sakai, 1976; Becker and Türkay, 2017; de Gier and Becker, 2020). Pinnotherid crabs are known to affect host physiology through direct consumption of food particles, mechanical interference with gill function, and diversion of host energetic resources, potentially leading to reduced growth, condition, and reproductive output of the host bivalves (Christensen and McDermott, 1958; Sugiura et al., 1960).

Host specificity in pinnotherid crabs varies markedly among taxa. Some species exhibit strict host specialization, whereas others exploit a broader range of bivalve hosts depending on local availability and environmental conditions (Watanabe, 2013; Becker and Türkay, 2017). Such plasticity in host use has been documented repeatedly in Japanese waters, where several pinnotherid species have expanded or shifted host associations, including utilization of non-indigenous bivalves (Yamada et al., 2009; Itani et al., 2011). These observations suggest that host–parasite relationships in Pinnotheridae are dynamic and context dependent, and that undocumented host associations may still be widespread, particularly in managed or disturbed ecosystems.

The species complex commonly referred to as the “kagizume-pinno,” *Pinnotheres pholadis* sensu lato, has long been recognized as a symbiont of various bivalves in Japanese waters. Its larval development and early life history have been described in detail (Fukuda, 1983; Muraoka, 1979), indicating early acquisition of host-dependent life stages. Although *P. pholadis* and closely related taxa have been reported from several bivalve hosts, including clams and scallops of other genera, host records remain fragmentary and regionally biased (Takeda and Konishi, 1988; Marin, 2014; Kolpakova et al., 2025). Notably, despite the ecological and economic importance of the Japanese scallop *Mizuhopecten yessoensis*, its role as a host for pinnotherid crabs has not been formally documented.

*Mizuhopecten yessoensis* is one of the most important aquaculture species in northern Japan, particularly in Aomori Prefecture, where large-scale suspension culture has supported regional fisheries for decades. However, scallop production in Aomori has declined sharply in recent years. Government statistics and reports from fisheries cooperatives indicate that total scallop landings in the prefecture have decreased by more than 50 % over the past five years, with especially severe reductions since 2022 (Fisheries Agency of Japan, 2025; Aomori Prefectural Fisheries Cooperative Association, 2025). This decline has been attributed primarily to mass mortality events associated with elevated summer water temperatures, poor spat settlement, and reduced survival of juvenile and subadult scallops, compounded by additional stressors such as predation and disease outbreaks.

While environmental stressors such as high water temperature are considered the principal drivers of recent scallop losses, biotic factors that may exacerbate host vulnerability have received comparatively little attention. Parasitic and symbiotic organisms, including pinnotherid crabs, have the potential to reduce host condition and resilience, particularly during early life stages when energetic reserves are limited. Experimental and observational studies on other bivalves have demonstrated that pinnotherid infestation can significantly reduce soft tissue mass and overall condition of the host (Christensen and McDermott, 1958; Sugiura et al., 1960). Such sublethal effects may not directly cause mortality but can increase susceptibility to additional stressors, including thermal stress and disease. Although mortality was not quantified in this study, reduced somatic condition in juveniles may plausibly reduce resilience to thermal stress and disease.

In this context, understanding the occurrence and impact of pinnotherid crabs in Japanese scallops is timely and relevant. If pinnotherid parasitism affects growth or somatic condition of *M. yessoensis*, particularly during juvenile stages, it could contribute indirectly to reduced production by slowing growth, increasing size variability, or amplifying mortality under adverse environmental conditions. Despite these potential implications, quantitative assessments of pinnotherid infestation in scallop aquaculture systems are lacking.

The present study documents the first confirmed occurrence of pinnotherid crabs inhabiting the mantle cavity of *M*. *yessoensis* in Mutsu Bay, northern Japan. Using field observations and quantitative analyses, we evaluate parasitism prevalence across developmental stages, examine host–parasite size relationships, and assess stage-dependent effects of parasitism on shell growth and somatic condition. By integrating these findings with recent declines in scallop production, this study provides new insights into the ecological role of pinnotherid crabs in a managed bivalve population and highlights parasitism as a previously overlooked factor that may interact with environmental stressors to influence scallop performance and aquaculture sustainability.

## 2 Materials and methods

### 2.1 Study area and sampling

**Fig. 1:**
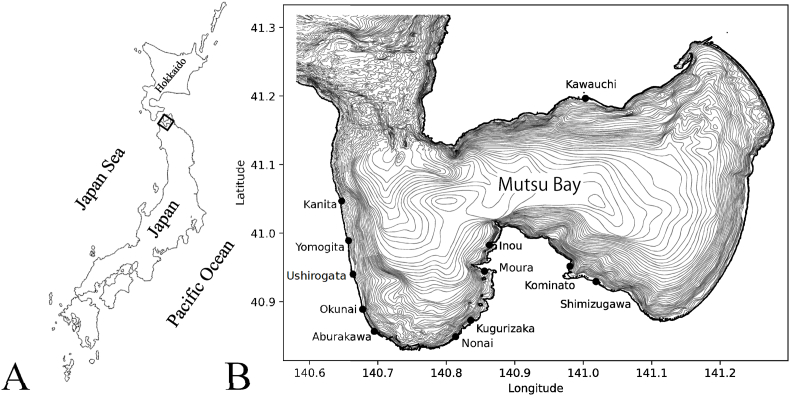
Bathymetric map of Mutsu Bay, northern Japan, showing sampling sites of the Japanese scallop *Mizuhopecten yessoensis*. Depth contours are shown at intervals between 10 and 200 m. Fig (A) indicates the location of Mutsu Bay within Japan, and fig (B) shows an enlarged view of the bay. Filled circles indicate scallop sampling sites. Bathymetry, coastline, and low-tide line in fig (B) are based on the M5000-series bathymetric dataset provided by the Japan Hydrographic Association.

Scallops were collected from multiple localities in Mutsu Bay, Aomori Prefecture, northern Japan (Fig. 1). Sampling was conducted between March 2024 and November 2025 and included both wild-caught and cultured Japanese scallops *Mizuhopecten yessoensis*. Details of sampling dates, localities, water depths, developmental stages, and numbers of scallops examined are summarized in Table 1.

**Table 1:** Sampling overview of *Mizuhopecten yessoensis* and associated pinnotherid crabs in Mutsu Bay. The table lists sampling dates, localities, numbers of scallops examined, mean shell length (±SD), developmental stage, and, where applicable, the number of parasitic crabs and their mean carapace length (CL) and carapace width (CW) (±SD). CL represents the anterior–posterior carapace dimension, whereas CW represents the maximum lateral width and was used for host–parasite size constraint analyses. ND indicates no data available.

Date	Locality	Scallop			Crab					
		Number of individuals	Mean shell length (mm) ± SD	Stage	Number of individuals	Sex			Mean carapace width (mm) ± SD	Mean carapace length (mm) ± SD
						Females	Male	Unknown		
30/10/2025	kominato	37	16.02 ± 2.43	juvenile	27	7	5	15	1.54 ± 0.47	2.44 ± 0.91
06/11/2025	kugurizaka	100	20.19 ± 2.41	juvenile	16	7	6	3	2.23 ± 0.55	2.37 ± 0.62
10/11/2025	ushirogata	84	14.55 ± 1.52	juvenile	35	13	11	11	1.59 ± 0.42	1.74 ± 0.46
25/11/2025	moura	20	26.85 ± 2.54	juvenile	20	13	5	2	2.20 ± 0.46	2.34 ± 0.51
19/05/2025	ushirogata	50	61.27 ± 7.98	subadult	14	ND	ND	ND	ND	ND
19/05/2025	shimizugawa	40	54.96 ± 14.38	subadult	8	ND	ND	ND	ND	ND
20/05/2025	inou	50	59.66 ± 9.84	subadult	18	ND	ND	ND	ND	ND
20/05/2025	kanita	50	57.54 ± 8.35	subadult	10	ND	ND	ND	ND	ND
20/05/2025	Nonai	50	60.03 ± 8.96	subadult	13	ND	ND	ND	ND	ND
21/05/2025	aburakawa	50	61.14 ± 6.49	subadult	7	4	3	0	3.25 ± 0.29	3.46 ± 0.29
21/05/2025	moura	50	57.90 ± 8.40	subadult	15	4	11	0	3.39 ± 0.37	3.59 ± 0.40
21/05/2025	yomogita	50	67.89 ± 9.54	subadult	13	8	5	0	3.82 ± 0.36	4.02 ± 0.33
21/05/2025	noheji	50	64.39 ± 7.55	subadult	11	7	4	0	3.28 ± 0.58	3.29 ± 0.56
22/05/2025	kominato	50	56.84 ± 8.97	subadult	28	16	12	0	2.99 ± 0.53	3.14 ± 0.56
23/05/2025	okunai	50	60.02 ± 5.42	subadult	22	15	7	0	3.25 ± 0.57	3.45 ± 0.58
28/05/2025	kugurizaka	50	71.83 ± 10.54	subadult	25	11	14	0	3.85 ± 0.93	4.00 ± 0.92
11/06/2025	kawauchi	50	76.09 ± 9.42	subadult	8	4	4	0	3.83 ± 0.69	4.03 ± 0.66

Scallops were obtained by hand during routine handling of aquaculture facilities or experimental stocks. Cultured scallops included juvenile, subadult, and one-year-old individuals reared using standard suspension culture methods, including ear-hanging and pearl-net systems. In some cases, scallops temporarily held at experimental facilities or research piers were also examined. Immediately after collection, scallops were opened and inspected for the presence of pinnotherid crabs within the mantle cavity.

### 2.2 Detection and identification of pinnotherid crabs

The mantle cavity of each scallop was carefully examined visually, with particular attention to the area between the mantle lobes and near the adductor muscle. When present, pinnotherid crabs were gently removed using forceps. The number of crabs per host scallop was recorded as parasite load (0, 1, or ≥2).

Crabs were provisionally identified as *Pinnotheres pholadis* De Gier and Becker, 2020 sensu lato based on external morphology, following published taxonomic descriptions of pinnotherid crabs from Japanese waters (Sakai, 1976; Takeda and Konishi, 1988; Becker and Türkay, 2017). Diagnostic characters used for identification included the weakly calcified, subcircular carapace; the well-developed chelipeds with curved dactyli; and marked sexual dimorphism in abdominal morphology, with females possessing a broad, rounded abdomen and males a narrow, triangular abdomen.

The observed morphological traits and the association with bivalve hosts are consistent with the traditional concept of the “kagizume-pinno” (*P. pholadis* sensu lato) commonly reported from Japanese coastal waters. Given recent recognition that *P. pholadis* represents a species complex with unresolved taxonomic boundaries, the present identification should be regarded as provisional.

Megalopa-stage individuals were identified based on larval morphology and excluded from analyses of crab size and sex. Recovered crabs were photographed for documentation, and representative individuals, including mature females and ovigerous specimens, were preserved as voucher specimens. Voucher specimens are deposited in the University Museum, The University of Tokyo (UMUT), Japan, to ensure long-term accessibility for future taxonomic and molecular studies (UMUT RA34570–RA34572).

### 2.3 Host measurements

For each scallop, shell length (SL, mm) was measured as the maximum anterior–posterior distance using digital calipers. Total wet weight and soft tissue wet weight were recorded to the nearest 0.01 g after blotting excess water. Soft tissue index (STI) was calculated as the ratio of soft tissue wet weight to total wet weight and used as an indicator of somatic condition.

Scallops were classified into developmental stages (juvenile or subadult) based on shell length and culture history (Table 1).

### 2.4 Crab measurements

For each pinnotherid crab, carapace length (CL, mm) and carapace width (CW, mm) were measured using digital calipers under a stereomicroscope. Sex was determined based on abdominal morphology. For hosts harboring multiple crabs, measurements were recorded separately for each individual crab.

### 2.5 Statistical analysis

Parasitism prevalence between developmental stages (juvenile vs. subadult) was compared using Fisher's exact test. Sex differences in crab size were evaluated using Welch's *t*-test. This test was conducted only to justify pooling sexes in subsequent host–parasite size analyses.

Effects of pinnotherid parasitism on scallop shell length were examined using a general linear model (GLM) with shell length as the response variable and developmental stage and parasitism status (parasitized vs. non-parasitized) as fixed factors.

The effects of pinnotherid parasitism on host somatic condition were examined using a GLM with soft tissue index (STI) as the response variable. Developmental stage (juvenile, subadult) and parasite load (0, 1, or ≥2 crabs per host) were included as fixed factors, shell length was included as a covariate, and the interaction between developmental stage and shell length was tested to assess stage-specific size effects. Parasite load was treated as a categorical factor, with non-parasitized scallops (0 crabs) used as the reference level.

Model assumptions of normality and homoscedasticity were evaluated by visual inspection of residual plots and quantile–quantile plots. Model-predicted means and 95 % confidence intervals were calculated at the overall mean shell length and visualized to illustrate size-adjusted effects of parasitism on host condition.

Host–parasite size constraints were examined using quantile regression. The upper boundary of the relationship between scallop shell length and crab carapace width was estimated using the 95th quantile, allowing assessment of maximum parasite size as a function of host size. Significance of regression coefficients was evaluated using standard quantile regression inference.

All statistical analyses were conducted using Python 3.11. Statistical modeling and hypothesis testing were performed using statsmodels 0.14.6. Statistical significance was assessed at α = 0.05. GLMs were fitted assuming Gaussian errors with an identity link (i.e., standard linear models).

## 3 Results

### 3.1 Identification of pinnotherid crabs

**Fig. 2:**
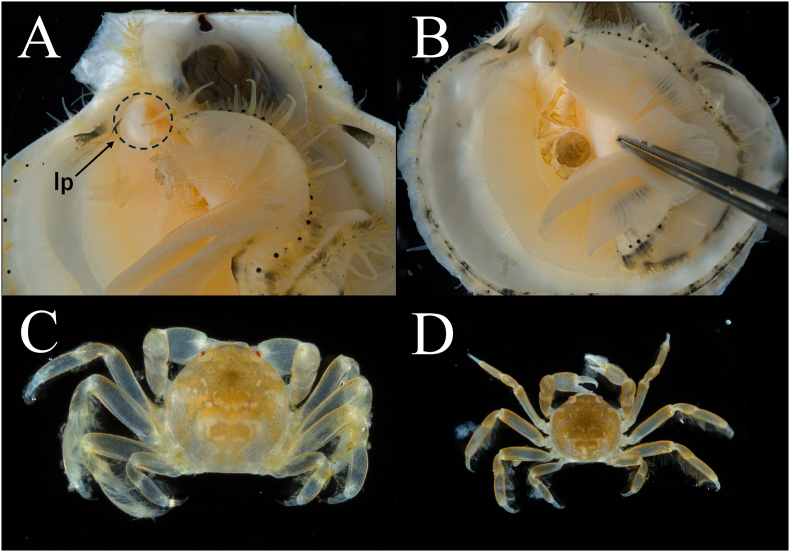
Two *Pinnotheres pholadis* inhabiting the mantle cavity of a single subadult Japanese scallop *Mizuhopecten yessoensis*. Figures A and C show the first crab individual (carapace length, CL = 3.42 mm), and Figures B and D show the second individual (CL = 5.18 mm). The host scallop (shell length, SL = 51.9 mm) was collected by hand on 29 March 2024 at a depth of 35 m off Moura, Hiranai Town, Mutsu Bay, northern Japan. The dashed circle in figure A indicates the labial palps (lp), a key feeding structure involved in particle sorting and transport toward the mouth. The illustrated specimens are deposited in the University Museum, The University of Tokyo (UMUT) as UMUT RA34570 (Fig. C) and UMUT RA34571 (Fig. D).

**Fig. 3:**
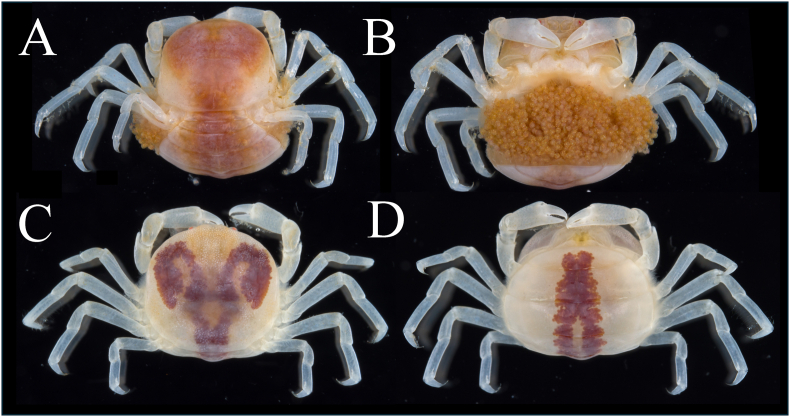
Female *Pinnotheres pholadis* parasitizing the mantle cavity of the Japanese scallop *Mizuhopecten yessoensis*. Figures A–B show a female collected by hand on 29 July 2025 at a depth of 1 m off Mogiura (carapace length, CL = 7.49 mm; carapace width, CW = 7.88 mm), from a subadult scallop (shell length, SL = 66.12 mm) temporarily held at the pier of the research institute. Figures C–D show a mature ovigerous female collected on 4 September 2025 at a depth of 18 m (mainline depth 6 m) off Kominato (CL = 7.15 mm; CW = 7.78 mm), parasitizing a cultured subadult scallop (SL = 76.25 mm) reared in a pearl net. The female in figures C–D carried eggs at a late developmental stage immediately prior to hatching. The specimen illustrated in Figures A–B is deposited in the University Museum, The University of Tokyo (UMUT) under accession number UMUT RA34571.

All parasitic crabs recovered from the mantle cavity of *Mizuhopecten yessoensis* shared a consistent suite of morphological characters diagnostic of pinnotherid crabs of the *Pinnotheres pholadis* species complex. Examined individuals exhibited a weakly calcified, subcircular carapace, relatively long and slender walking legs, and well-developed chelipeds with distinctly curved dactyli. Clear sexual dimorphism was observed in abdominal morphology, with females possessing a broad, rounded abdomen adapted for egg brooding, whereas males had a narrow, triangular abdomen. In some cases, multiple pinnotherid crabs were recovered simultaneously from the mantle cavity of a single host scallop (Fig. 2). Ovigerous females carrying late-stage eggs were frequently recorded within host scallops (Fig. 3), confirming sexual maturity and successful reproduction in the examined population.

Megalopa-stage individuals, when present, were readily distinguished by larval morphology and excluded from adult morphometric and sex-based analyses. Although the observed morphology and host association are fully consistent with the traditional concept of the “kagizume-pinno,” *Pinnotheres pholadis* sensu lato, recent taxonomic studies indicate that this taxon represents a species complex with unresolved boundaries. Accordingly, species-level identification in the present study is treated as provisional, pending future integrative taxonomic analyses combining detailed morphology and molecular data.

### 3.2 Occurrence and host association of pinnotherid crabs in Japanese scallops

A total of 881 Japanese scallops *Mizuhopecten yessoensis* were examined from multiple aquaculture sites in Mutsu Bay, Aomori Prefecture, between March 2024 and November 2025 (Table 1).

Pinnotherid crabs were found inhabiting the mantle cavity of scallops at several localities. When present, hosts contained one or two crab individuals, including both males and females. Sex could be determined for all crabs recovered from subadult scallops, whereas a proportion of crabs from juvenile scallops remained unsexed due to their small body size (Table 1). Among sexed individuals, females were more frequently recovered than males, and ovigerous females were commonly observed, indicating that reproduction occurred within the host scallops. No external signs of infestation were apparent prior to opening the shells.

The numbers of scallops examined, parasitized individuals, recovered pinnotherid crabs, and the absolute numbers of female, male, and unsexed crabs together with sampling dates, localities, water depths, and developmental stages, are summarized in Table 1. These observations confirm that pinnotherid infestation was widespread within the surveyed aquaculture system and occurred across developmental stages.

### 3.3 Parasitism prevalence by developmental stage

A total of 241 juvenile scallops and 640 subadult scallops were examined for pinnotherid parasitism. Parasitism prevalence was slightly higher in juvenile scallops (32.8 %) than in subadults (27.3 %); however, this difference was not statistically significant (Fisher's exact test, p = 0.114). These results indicate that pinnotherid infestation occurs across developmental stages without a strong stage-specific bias in prevalence.

### 3.4 Effects of parasitism on somatic condition

STI varied with parasite load and developmental stage after accounting for shell length (GLM, parasite load: p < 0.001; developmental stage: p < 0.001). The interaction between developmental stage and shell length showed a marginal effect on STI (stage × shell length, p = 0.081), indicating that the relationship between host size and somatic condition differed weakly between juvenile and subadult scallops.

### 3.5 Size-adjusted effects of parasite load on somatic condition

**Fig. 4:**
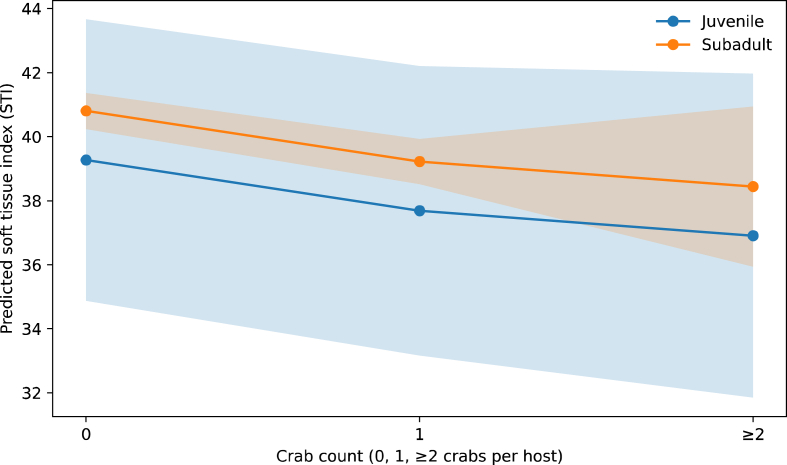
Predicted soft tissue index (STI) of juvenile and subadult Japanese scallops *Mizuhopecten yessoensis* in relation to pinnotherid crab count (0, 1, or ≥2 crabs per host). Predictions are derived from a general linear model (GLM) with shell length included as a covariate and are shown at the overall mean shell length (50.28 mm). Shaded areas indicate 95 % confidence intervals.

After controlling for shell length, parasite load had a significant negative effect on scallop somatic condition. Predicted STI values decreased with increasing numbers of pinnotherid crabs per host in juvenile scallops, while corresponding effects were weak or absent in subadults. These results indicate that reductions in somatic condition associated with pinnotherid parasitism cannot be explained solely by host size differences, but reflect a direct negative impact of parasitism that is most pronounced at early developmental stages. These size-adjusted patterns are illustrated by the model-predicted values shown in Fig. 4.

### 3.6 Effects of parasitism on scallop shell growth

Parasitism had a clear negative effect on shell growth in juvenile scallops but not in subadults. Parasitized juvenile scallops exhibited significantly shorter shell lengths than non-parasitized individuals (GLM, p = 1.25 × 10^−7^). In contrast, no significant difference in shell length was detected between parasitized and non-parasitized subadult scallops (GLM, p = 0.152).

### 3.7 Size characteristics of parasitic crabs

Carapace width did not differ significantly between male and female pinnotherid crabs (Welch's *t*-test, p = 0.18), and data from both sexes were therefore pooled for subsequent analyses of host–parasite size relationships.

### 3.8 Host–parasite size constraint

**Fig. 5:**
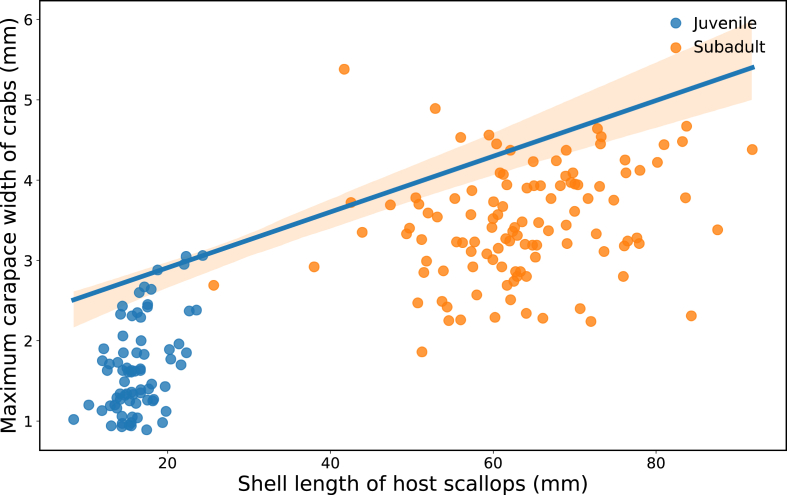
Size constraint between shell length of host scallops (SL, mm) and the maximum size of pea crabs expressed as carapace width (CW, mm) in parasitized Japanese scallops *Mizuhopecten yessoensis*. Each point represents a single host scallop plotted against the maximum CW of crabs inhabiting that host, thereby avoiding pseudoreplication. Juvenile hosts (October–November) are shown in blue, and subadult hosts (May–June) in orange. The upper boundary of the relationship was estimated using 95 % quantile regression: CW = 1.85 + 0.0347 × SL (p < 0.001).

Quantile regression analysis revealed a clear upper boundary in the relationship between host scallop shell length and maximum crab size (Fig. 5). The 95 % quantile regression model (Carapace width = 1.85 + 0.0347 × Shell length) showed significant intercept and slope values (p < 0.001), indicating a strong host-size limitation whereby larger scallops can host proportionally larger pinnotherid crabs.

## 4 Discussion

### 4.1 First host record and successful establishment in Japanese scallops

The present study provides the first documented evidence that pinnotherid crabs parasitize the Japanese scallop *Mizuhopecten yessoensis*. Pinnotherid crabs are well known for their symbiotic associations with a wide variety of bivalve hosts worldwide (De Gier and Becker, 2020–1850; Sakai, 1976; Becker and Türkay, 2017; de Gier and Becker, 2020), yet no previous study has formally recorded their occurrence in *M. yessoensis*, despite its long-standing economic and ecological importance in northern Japan.

The detection of pinnotherid crabs across multiple localities within Mutsu Bay indicates that this association is not accidental but established at the population level. Similar expansions or shifts in host use have been reported for other pinnotherid species in Japanese waters, including utilization of both native and non-indigenous bivalves (Yamada et al., 2009; Itani et al., 2011), suggesting that host associations in this family can be flexible and context dependent.

The frequent occurrence of mature and ovigerous females within scallop hosts further indicates that *M. yessoensis* functions as a suitable reproductive host. Completion of the reproductive cycle within a host is generally regarded as a key criterion distinguishing true parasitism from transient commensalism (Christensen and McDermott, 1958; Becker and Türkay, 2017). Our observations therefore support the interpretation that pinnotherid crabs are not merely incidental occupants but are successfully established parasites in Japanese scallops.

### 4.2 Parasitism prevalence and developmental-stage neutrality

Parasitism prevalence did not differ significantly between juvenile and subadult scallops, indicating that pinnotherid infestation occurs across developmental stages without strong stage-specific bias in occurrence. This pattern suggests that encounter rates between host and parasite, or the initial establishment of crabs within the mantle cavity, are broadly similar once scallops reach a minimum size threshold capable of accommodating a crab.

Comparable patterns have been reported in other bivalve–pinnotherid systems, where infestation probability is often weakly related to host age or size once hosts exceed a critical minimum dimension (Christensen and McDermott, 1958; Sugiura et al., 1960).

Importantly, the absence of a strong stage effect on prevalence contrasts sharply with the pronounced stage-dependent impacts of parasitism on host performance detected in this study. This decoupling between infestation probability and physiological impact highlights the limitation of prevalence-based assessments alone and emphasizes the importance of evaluating host condition and growth consequences across life stages.

### 4.3 Stage-dependent impacts on growth and somatic condition

Despite similar prevalence across stages, pinnotherid parasitism had markedly different effects on host performance. Parasitized juvenile scallops exhibited significantly reduced shell growth and soft tissue index, whereas no statistically significant effects were detected in subadults. This stage-dependent vulnerability is consistent with the general expectation that younger or smaller hosts possess lower energetic reserves and reduced capacity to compensate for parasitic stress.

Previous studies on other bivalves have shown that pinnotherid crabs can reduce host feeding efficiency, divert ingested food, and mechanically interfere with gill function, leading to reduced somatic condition and growth (Christensen and McDermott, 1958; Sugiura et al., 1960). Such effects are likely to be disproportionately severe in early life stages, when energetic demands for shell accretion and tissue growth are high.

In contrast, subadult scallops may partially compensate for parasitic demands through higher absolute feeding rates or greater storage capacity. Similar ontogenetic shifts in tolerance to parasitism have been reported in other host–parasite systems, where sublethal effects are strongest during early developmental stages and become attenuated as hosts grow larger.

### 4.4 Size-adjusted effects and direct parasitic costs

Results from size-adjusted statistical models demonstrate that parasite load significantly reduced soft tissue index even after accounting for host shell length, providing strong evidence for a direct physiological cost of pinnotherid parasitism. This finding indicates that the observed reduction in somatic condition cannot be explained solely by size differences between parasitized and non-parasitized scallops.

Direct energetic costs imposed by pinnotherid crabs have been documented previously, including food theft, damage to gill tissues, and chronic energetic diversion (Christensen and McDermott, 1958; Sugiura et al., 1960; de Gier and Becker, 2020). The present results extend these findings to *M. yessoensis* and demonstrate that such costs are detectable even at relatively low parasite loads.

Notably, parasite load exerted a measurable effect on somatic condition across developmental stages, suggesting that even low-intensity infestations may reduce host resilience. Such sublethal impacts may not directly cause mortality but could increase susceptibility to additional stressors, including elevated temperature or disease, particularly under aquaculture conditions.

### 4.5 Host–parasite size constraints and infestation limits

The clear upper boundary in the relationship between host shell length and maximum crab size indicates a strong host-size constraint on pinnotherid infestation. Similar size-dependent constraints have been reported in other pinnotherid–bivalve associations and are generally interpreted as reflecting spatial limitation within the mantle cavity and mechanical constraints on crab growth (Christensen and McDermott, 1958; Becker and Türkay, 2017).

The marginal stage × size interaction detected in the size-adjusted models suggests that increases in shell length translate into somatic benefits more strongly in juvenile scallops than in subadults. Although this interaction did not reach conventional levels of statistical significance, the trend is biologically meaningful in the context of ontogenetic shifts in energy allocation and tolerance to parasitic stress. Juvenile scallops, which invest heavily in shell accretion and tissue growth, may therefore experience disproportionate energetic costs when parasitized, whereas subadults with greater absolute feeding capacity and energy reserves may partially buffer these costs as body size increases.

In several examined hosts, pinnotherid crabs were often found positioned near the labial palps, immediately upstream of the oral region (Fig. 2), although microhabitat use was not quantified systematically. The labial palps represent a key sorting structure in the feeding pathway, where particles captured by the gills are processed and directed toward the mouth. Positioning near this region may facilitate interception of food particles and/or interfere with particle sorting and transport, thereby providing a plausible mechanistic link to reduced host condition.

This spatial preference provides a functional mechanism linking early establishment and host–parasite size constraints. One plausible scenario is that early infection of small hosts may allow crabs to secure this high-value feeding niche, with subsequent crab growth tracking host growth as mantle cavity volume and feeding throughput increase. However, ultimate crab size remains constrained by the physical and functional limits of the labial palp region, offering a plausible explanation for the strong upper boundary observed in the host–parasite size relationship (Fig. 5).

Smaller scallops hosting relatively large crabs may experience disproportionately high energetic and mechanical stress, providing a plausible explanation for the severe condition loss observed in parasitized juvenile scallops despite comparable infestation prevalence across stages.

Such host–parasite size constraints may also influence parasite life-history strategies, potentially favoring early establishment in smaller hosts followed by growth tracking host size. Understanding these constraints is therefore important for predicting both parasite dynamics and host vulnerability.

### 4.6 Implications for scallop aquaculture and managed ecosystems

The Japanese scallop is a cornerstone species for aquaculture in northern Japan, and early-life performance is critical for production success. Recent declines in scallop landings in Aomori Prefecture have been attributed primarily to environmental stressors such as elevated water temperature and mass mortality events (Fisheries Agency of Japan, 2025; Aomori Prefectural Fisheries Cooperative Association, 2025).

The present results suggest that pinnotherid parasitism represents an additional, previously overlooked biotic stressor that can substantially reduce growth and somatic condition during the juvenile stage. Although parasitism prevalence was not higher in juvenile scallops, the disproportionate impact at this stage indicates that even moderate infestation levels may contribute indirectly to reduced production by slowing growth, increasing size variability, or amplifying mortality under adverse conditions.

From a management perspective, these findings highlight the importance of incorporating parasitic interactions into assessments of scallop health and resilience. Early detection and monitoring of pinnotherid infestation may be particularly important during juvenile stages, when hosts appear most vulnerable.

Although mortality was not quantified in this study, reduced somatic condition in juvenile scallops may plausibly lower tolerance to additional stressors, including elevated temperature and disease, thereby increasing the likelihood of mortality during summer warming events. In the context of recent mass mortality events reported in Aomori Prefecture, this possibility underscores the need to integrate parasitism into health monitoring during early life stages.

### 4.7 Future perspectives and taxonomic considerations

While this study focused on ecological impacts, precise taxonomic identification of the pinnotherid crabs remains an important next step. Molecular analyses, including COI sequencing, will help clarify species identity and facilitate comparisons with pinnotherid populations reported from other hosts and regions (Becker and Türkay, 2017; de Gier and Becker, 2020).

Integrating molecular taxonomy with the ecological framework established here will improve our understanding of host specificity, host-switching potential, and parasite dynamics in both natural and cultured systems, and will contribute to more comprehensive management strategies for bivalve aquaculture under increasing environmental stress.

Given that *Pinnotheres pholadis* represents a morphologically conserved species complex, unresolved taxonomic boundaries may obscure cryptic host-associated lineages, and the present record from *Mizuhopecten yessoensis* raises the possibility that host expansion or host switching has occurred within this complex in response to local ecological conditions.

### 4.8 Parasitism versus commensalism: functional interpretation of the host–crab interaction

Although pinnotherid crabs have historically been described as commensals in some bivalve hosts, accumulating evidence suggests that their ecological role spans a continuum from commensalism to true parasitism (Christensen and McDermott, 1958; Becker and Türkay, 2017; de Gier and Becker, 2020). In the present study, the interaction between pinnotherid crabs and the Japanese scallop *Mizuhopecten yessoensis* is best interpreted as functional parasitism rather than benign commensalism. This conclusion is supported by multiple lines of quantitative evidence. First, parasitized scallops, particularly at the juvenile stage, exhibited significantly reduced shell growth and soft tissue index compared with non-parasitized individuals. Second, size-adjusted statistical models demonstrated that parasite load exerted a significant negative effect on somatic condition even after accounting for host shell length, indicating a direct physiological cost of infestation independent of host size. Third, the frequent occurrence of mature and ovigerous females within host scallops indicates that the crab completes its reproductive cycle inside the host, relying on the host not only as shelter but also as a feeding and reproductive habitat. Importantly, parasitism prevalence did not differ between developmental stages, whereas impacts were strongly stage dependent, suggesting that early-life stages of the host are particularly vulnerable to energetic diversion and mechanical interference caused by the parasite. Taken together, these findings demonstrate that pinnotherid crabs impose measurable fitness-related costs on *M. yessoensis* and therefore function as true parasites in this system, even if the interaction may appear weak or context dependent in larger, more resilient hosts. This functional perspective provides a practical framework for evaluating pinnotherid host impacts without relying on historical labels that may obscure ecologically meaningful effects.

## CRediT authorship contribution statement

**Tomoyasu Yamazaki:** Writing – review & editing, Writing – original draft, Visualization, Resources, Project administration, Methodology, Formal analysis, Data curation, Conceptualization. **Kenji Odani:** Methodology, Investigation, Funding acquisition, Data curation. **Ryo Nakayama:** Formal analysis, Data curation. **Tetsuya Watanabe:** Methodology. **Souichirou Kawai:** Methodology.

## Conflict of interest

The authors declare that they have no known competing financial interests or personal relationships, including employment, consultancies, stock ownership, honoraria, paid expert testimony, or patent applications, that could have appeared to influence the work reported in this paper.
